# Supplementary material for: Facilitation of a free-roaming apex predator in working lands: evaluating factors that influence leopard spatial dynamics and prey availability in a South African biodiversity hotspot
Source: PeerJ. 2023 Jan 25;11:e14575. doi: 10.7717/peerj.14575 (PMC9884037; doi:10.7717/peerj.14575)
Supplement: Supplemental Information 1 [file peerj-11-14575-s001.docx]

**Supplementary material**

Table S1: Site covariates for modelling space-use and detection probabilities of leopards and their main prey species in the Cederberg and Piketberg.

| Covariate^*^ | Description | Category | Source |
| --- | --- | --- | --- |
| Altitude | Numerical, measured in metres above sea level (m.a.s.l.) | Environmental | GPS,  Google Earth |
| Vegetation Type (vegetation/veg) | Categorical, five classes (Fynbos, Karoo, Sandveld, Renosterveld, Riverine thicket) | Environmental | Site inspection, Mucina & Rutherford (2006) |
| Vegetation Age  (veg age) | Categorical, four classes in years (<1, 1-3, 3-10, >10) | Environmental | Site inspection, Fire history (GCFPA) |
| Classification of nearest water source (water source) | Categorical, either seasonal (stream/pool) or permanent (river/dam/spring) | Environmental | Site inspection |
| Minimum distance to water resource  (water) | Numerical, measured in metres (m) | Environmental | Site inspection, Google Earth |
| Distance to the nearest road  (road) | Numerical, measured in metres (m) | Anthropogenic | Site inspection, Google Earth |
| Distance to the nearest human habitation (habitation) | Numerical, measured in metres (m) | Anthropogenic | Site inspection, Google Earth |
| Evidence of anthropogenic disturbance (disturbance) | Categorical, presence or absence (yes or no), including rubbish or frequented fireplaces | Anthropogenic | Site inspection, Camera-trap |
| Evidence of livestock (livestock) | Categorical, presence or absence (yes or no), including tracks or droppings | Anthropogenic | Site inspection, Camera-trap |
| Evidence of hunting (hunting) | Categorical, presence or absence (yes or no), including snares or other traps | Anthropogenic | Site inspection, Camera-trap |
| Main prey species (prey) | Numerical, relative abundance index (RAI) aggregated for baboon, duiker, grysbok, hyrax, klipspringer, porcupine, rhebok | Biotic | Camera-trap |
| Sympatric meso-predators (carnivore/caracal) | Numerical, RAI for caracal and/or black-backed jackal | Biotic | Camera-trap |
| Leopard | Numerical, RAI | Biotic | Camera-trap |

* Rationale: (Pitman et al., 2017)

Table S2: Terrestrial mammals > 0.5 kg (i.e. potential natural leopard prey species) recorded in the Cederberg and Piketberg. Relative abundance indices for each species in each community are indicated in parentheses. Species considered as main prey species for leopards in the Western Cape are underlined. Y indicates that species were captured in each area whereas N means that they were not.

| Species | | Trophic level | Mean weight (kg)^†^ | Cederberg | Piketberg |
| --- | --- | --- | --- | --- | --- |
| Aardvark | *Orycteropus afer* | Insectivore | 55 | Y (0.41) | Y (1.41) |
| Aardwolf | *Proteles cristata* | Insectivore | 8.5 | Y (0.12) | Y (0.94) |
| African wild cat | *Felis silvestris lybica* | Carnivore | 4.3 | Y (0.74) | Y (1.02) |
| Bat-eared fox | *Otocyon megalotis* | Insectivore | 4 | Y (0.13) | Y (0.1) |
| Black-backed jackal | *Canis mesomelas* | Carnivore | 9 | Y (0.19) | N |
| Blue wildebeest | *Connochaetes taurinus* | Herbivore | 215 | Y (0.02) | N |
| Cape fox | *Vulpes chama* | Carnivore | 3 | N | Y (0.08) |
| Cape grysbok | *Raphicerus melanotis* | Herbivore | 10 | Y (0.8) | Y (7.91) |
| Cape mountain zebra | *Equus zebra zebra* | Herbivore | 255 | Y (1.17) | Y (0.18) |
| Caracal | *Caracal caracal* | Carnivore | 15 | Y (0.67) | Y (1.37) |
| Chacma baboon | *Papio ursinus* | Omnivore | 24 | Y (6.01) | Y (9.46) |
| Common duiker | *Sylvicapra grimmia* | Herbivore | 20 | Y (1.45) | Y (3.9) |
| Eland | *Taurotragus oryx* | Herbivore | 700 | Y (0.37) | Y (0.02) |
| Gemsbok | *Oryx gazella* | Herbivore | 225 | Y (2.37) | N |
| Grey rhebok | *Pelea capreolus* | Herbivore | 20 | Y (0.84) | Y (0.14) |
| Hares & rabbits^*^ | *Lagomorpha sp.* | Herbivore | 2.2 | Y (0.58) | Y (3.15) |
| Honey badger | *Mellivora capensis* | Carnivore | 11 | Y (0.59) | Y (0.89) |
| Klipspringer | *Oreotragus oreotragus* | Herbivore | 13 | Y (3.56) | Y (2.41) |
| Kudu | *Tragelaphus strepsicerus* | Herbivore | 220 | N | Y (0.03) |
| Large grey mongoose | *Herpestes ichneumon* | Carnivore | 3.2 | N | Y (0.26) |
| Large spotted genet | *Genetta tigrina* | Carnivore | 2.5 | Y (0.49) | Y (0.94) |
| Leopard | *Panthera pardus* | Carnivore | 35 | Y (3.52) | Y (4.65) |
| Porcupine | *Hystrix africaeaustralis* | Herbivore | 15 | Y (3.36) | Y (8.63) |
| Red hartebeest | *Alcelaphus buselaphus caama* | Herbivore | 135 | Y (0.13) | N |
| Rock hyrax | *Procavia capensis* | Herbivore | 3.7 | Y (4.05) | Y (3.16) |
| Small grey mongoose | *Galerella pulverulenta* | Carnivore | 0.8 | Y (0.64) | Y (0.62) |
| Small spotted genet | *Genetta genetta* | Carnivore | 2.1 | Y (0.12) | Y (0.11) |
| Steenbok | *Raphicerus campestris* | Herbivore | 11 | Y (0.02) | Y (0.05) |
| Striped polecat | *Ictonyx striatus* | Carnivore | 1 | Y (0.09) | Y (0.08) |
| Water mongoose | *Atilax paludinosus* | Carnivore | 3.5 | Y (0.07) | N |

† (Stuart & Stuart, 2015)

* potentially included - Cape hare (*Lepus capensis),* Hewitt’s red rock rabbit (*Pronolagus saundersiae*), and scrub hare (*Lepus saxatilis*)

Table S3: Goodness-of-fit results from the global occupancy models for each species in each study area (i.e. Cederberg and Piketberg), using different collapsing periods. The chi-square probability (χ^2^p) and over dispersion statistic (ĉ) were used to choose the best-fit model for each species in each study area and is highlighted in bold. Covariates set for space-use and detection modelling for each study area are stipulated in the variables column.

| **Species** | **Sampling periods (days)** | **Cederberg** | | |  | **Piketberg** | | |
| --- | --- | --- | --- | --- | --- | --- | --- | --- |
|  |  | **ĉ** | **χ^2^p** | **Variables** |  | **ĉ** | **χ^2^p** | **Variables** |
| **Leopard** | 5 | 33.94 | 0.00 | altitude + road + habitation + water + livestock + disturbance + carnivore + water source + veg + prey |  | 366.33 | 0.00 | altitude + road + habitation + water + livestock + disturbance + caracal + veg age + water source + veg + prey |
|  | 6 | 96.96 | 0.00 |  |  | 52.6 | 0.00 |  |
|  | 7 | 5.8 | 0.01 |  |  | 1.97 | 0.05 |  |
|  | 8 | 3.23 | 0.00 |  |  | 3.24 | 0.01 |  |
|  | 9 | 2.52 | 0.01 |  |  | 1.51 | 0.02 |  |
|  | 10 | 1.65 | 0.01 |  |  | **1.2** | **0.08** |  |
|  | 11 | **1.08** | **0.22** |  |  | 0.76 | 0.87 |  |
| **Baboon** | 5 | 141949.70 | 0.00 | ĉ > 3 |  | 2.71 | 0.02 | altitude + road + habitation + water + livestock + disturbance + caracal + veg + leopard + water source + veg age |
|  | 6 | 1611.51 | 0.00 |  |  | 2.84 | 0.03 |  |
|  | 7 | 1704.62 | 0.00 |  |  | **1.26** | **0.16** |  |
|  | 8 | 455.83 | 0.00 |  |  | 1.49 | 0.11 |  |
|  | 9 | 126.15 | 0.00 |  |  | 1.87 | 0.01 |  |
|  | 10 | 36.05 | 0.00 |  |  | 1.39 | 0.02 |  |
|  | 11 | 48.13 | 0.00 |  |  | 1.54 | 0.03 |  |
|  |  |  |  |  |  |  |  |  |
| **Duiker** | 5 | 0.59 | 0.54 | altitude + road + habitation + water + livestock + disturbance + carnivore + water source + veg + veg age + leopard |  | **1.22** | **0.17** | altitude + road + habitation + water + livestock + disturbance + caracal + water source + veg + leopard + veg age |
|  | 6 | 1.4 | 0.17 |  |  | 2.48 | 0.05 |  |
|  | 7 | **1.03** | **0.52** |  |  | 2.04 | 0.04 |  |
|  | 8 | 1.09 | 0.50 |  |  | 1.85 | 0.05 |  |
|  | 9 | 1.06 | 0.49 |  |  | 2.08 | 0.00 |  |
|  | 10 | 0.53 | 0.61 |  |  | 0.82 | 0.91 |  |
|  | 11 | 0.52 | 0.60 |  |  | 1.72 | 0.04 |  |
| **Grysbok** | 5 | **1.22** | **0.24** | altitude + road + habitation + water + livestock + disturbance + carnivore + water source + veg + leopard |  | 1897.74 | 0.00 | altitude + road + habitation + water + livestock + disturbance + caracal + veg age + water source + veg + leopard |
|  | 6 | 8.12 | 0.00 |  |  | 28.03 | 0.00 |  |
|  | 7 | 1.50 | 0.05 |  |  | 12.82 | 0.00 |  |
|  | 8 | 11.72 | 0.00 |  |  | 1.25 | 0.14 |  |
|  | 9 | 1.62 | 0.01 |  |  | 1.33 | 0.04 |  |
|  | 10 | 1.81 | 0.00 |  |  | **1.07** | **0.32** |  |
|  | 11 | 1.69 | 0.04 |  |  | 1.11 | 0.26 |  |
| **Hyrax** | 5 | 3.45 | 0.01 | altitude + road + habitation + water + livestock + disturbance + carnivore + water source + veg + leopard |  | 10.28 | 0.00 | habitation + water + caracal + leopard |
|  | 6 | 80.6 | 0.00 |  |  | 0.54 | 0.63 |  |
|  | 7 | 607.31 | 0.00 |  |  | 2.35 | 0.06 |  |
|  | 8 | 1.26 | 0.12 |  |  | **1.09** | **0.28** |  |
|  | 9 | **1.10** | **0.16** |  |  | 0.86 | 0.43 |  |
|  | 10 | 1.38 | 0.05 |  |  | 0.86 | 0.43 |  |
|  | 11 | 0.86 | 0.64 |  |  | - | - |  |
| **Klipspringer** | 5 | 103.87 | 0.00 | altitude + road + habitation + water + livestock + disturbance + carnivore + water source + veg + leopard + veg age |  | 11.45 | 0.00 | altitude + road + habitation + water + livestock + disturbance + caracal + water source + veg + leopard |
|  | 6 | 21.59 | 0.00 |  |  | 3.32 | 0.02 |  |
|  | 7 | 1.64 | 0.03 |  |  | 3.18 | 0.00 |  |
|  | 8 | **1.36** | **0.04** |  |  | 1.79 | 0.05 |  |
|  | 9 | 0.76 | 0.83 |  |  | **1.07** | **0.35** |  |
|  | 10 | 0.89 | 0.71 |  |  | 0.72 | 0.95 |  |
|  | 11 | 0.59 | 0.99 |  |  | 1.10 | 0.20 |  |
| **Porcupine** | 5 | 18.95 | 0.00 | altitude + road + habitation + water + livestock + disturbance + carnivore + veg age + water source + veg + leopard |  | 5.97 | 0.00 | altitude + road + habitation + water + livestock + disturbance + caracal + water source + veg + leopard |
|  | 6 | 313.01 | 0.00 |  |  | 0.78 | 0.41 |  |
|  | 7 | 1.46 | 0.07 |  |  | 0.83 | 0.56 |  |
|  | 8 | **1.38** | **0.13** |  |  | 0.76 | 0.74 |  |
|  | 9 | 2.35 | 0.02 |  |  | 0.86 | 0.75 |  |
|  | 10 | 3.12 | 0.01 |  |  | **1.11** | **0.36** |  |
|  | 11 | 2.93 | 0.02 |  |  | 3.06 | 0.02 |  |
| **Rhebok** | 5 | 0.48 | 0.68 | χ^2^p < 0.05 |  | 0.27 | 0.51 | Models do not converge |
|  | 6 | 0.66 | 0.73 |  |  | 0.53 | 0.55 |  |
|  | 7 | 0.96 | 0.47 |  |  | 0.52 | 0.52 |  |
|  | 8 | 1.88 | 0.04 |  |  | **1.02** | **0.50** |  |
|  | 9 | 2.82 | 0.00 |  |  | 0.41 | 0.67 |  |
|  | 10 | **1.68** | **0.01** |  |  | 0.48 | 0.68 |  |
|  | 11 | 4.96 | 0.00 |  |  | 0.49 | 0.70 |  |

Table S4: Top ranked models (*w* > 0.10 and QAICc < 2) for each species for the Cederberg, using quasi-likelihood information criterion for small sample size (QAICc), delta QAICc (△QAICc), QAICc weight (QAICcwt) and the number of parameters (K).

| **Species** | **Model** | **K** | **QAICc** | **△QAICc** | **QAICcwt** |
| --- | --- | --- | --- | --- | --- |
| **Leopard** | Ψ(habitation + water source + prey) *p*(carnivore + altitude + water + prey) | 10 | 900.55 | 0.00 | 0.49 |
|  | Ψ(habitation + water source + prey + livestock) *p*(carnivore + altitude + water + prey) | 11 | 901.68 | 1.13 | 0.28 |
|  | Ψ(habitation + water source) *p*(carnivore + altitude + water + prey) | 9 | 902.03 | 1.49 | 0.23 |
|  |  |  |  |  |  |
| **Duiker** | Ψ(road + altitude) *p*(leopard+ altitude + water + vegetation age + vegetation + habitation + road) | 12 | 143.96 | 0.00 | 0.72 |
|  | Ψ(carnivore + water source) *p*(leopard+ altitude + water + vegetation age + vegetation + habitation + road) | 12 | 145.82 | 1.86 | 0.28 |
|  |  |  |  |  |  |
| **Grysbok** | Ψ(road + leopard + water source) *p*(vegetation + road + disturbance) | 9 | 303.00 | 0.00 | 0.16 |
|  | Ψ(road + leopard + water source + vegetation) *p*(vegetation + road + disturbance) | 10 | 3.03.03 | 0.03 | 0.16 |
|  | Ψ(road + water source) *p*(vegetation + road + disturbance) | 8 | 303.09 | 0.09 | 0.16 |
|  | Ψ(road) *p*(vegetation + road + disturbance) | 7 | 303.12 | 0.13 | 0.15 |
|  | Ψ(road + vegetation) *p*(vegetation + road + disturbance) | 8 | 303.72 | 0.72 | 0.11 |
|  | Ψ(road + leopard) *p*(vegetation + road + disturbance) | 8 | 303.74 | 0.74 | 0.11 |
|  | Ψ(road + altitude) *p*(vegetation + road + disturbance) | 8 | 304.69 | 1.69 | 0.07 |
|  | Ψ(road + water) *p*(vegetation + road + disturbance) | 8 | 304.70 | 1.70 | 0.07 |
|  |  |  |  |  |  |
| **Hyrax** | Ψ(vegetation + carnivore) *p*(vegetation + road + carnivore + altitude) | 9 | 682.39 | 0.00 | 0.36 |
|  | Ψ(vegetation + carnivore + water) *p*(vegetation + road + carnivore + altitude) | 10 | 683.00 | 0.62 | 0.27 |
|  | Ψ(vegetation + carnivore + leopard) *p*(vegetation + road + carnivore + altitude) | 10 | 683.42 | 1.03 | 0.22 |
|  | Ψ(vegetation + carnivore + disturbance) *p*(vegetation + road + carnivore + altitude) | 10 | 684.13 | 1.75 | 0.15 |
|  |  |  |  |  |  |
| **Klipspringer** | Ψ(altitude + water source) *p*(vegetation + carnivore + disturbance) | 8 | 735.25 | 0.00 | 0.32 |
|  | Ψ(altitude) *p*(vegetation + carnivore + disturbance) | 7 | 735.57 | 0.32 | 0.28 |
|  | Ψ(altitude + livestock) *p*(vegetation + carnivore + disturbance) | 8 | 736.80 | 1.55 | 0.15 |
|  | Ψ(altitude + water source + livestock) *p*(vegetation + carnivore + disturbance) | 9 | 737.08 | 1.83 | 0.13 |
|  | Ψ(water source) *p*(vegetation + carnivore + disturbance) | 7 | 737.19 | 1.94 | 0.12 |
|  |  |  |  |  |  |
| **Porcupine** | Ψ(vegetation age + water) *p*(vegetation + vegetation age + road + carnivore) | 9 | 526.56 | 0.00 | 0.38 |
|  | Ψ(vegetation age) *p*(vegetation + vegetation age + road + carnivore) | 8 | 527.10 | 0.55 | 0.29 |
|  | Ψ(vegetation age + water + leopard) *p*(vegetation + vegetation age + road + carnivore) | 10 | 528.02 | 1.46 | 0.18 |
|  | Ψ(water) *p*(vegetation + vegetation age + road + carnivore) | 8 | 528.43 | 1.87 | 0.15 |
|  |  |  |  |  |  |

Table S5: Model averaged covariate coefficient estimates (and 95% confidence intervals (CIs)) from the best approximating model from the analyses examining factors related to the space-use of species in the Cederberg. Bold estimates indicate that there was a strong association (CIs do not overlap zero) between the covariate and species occupancy/space-use (Ψ) or detection (*p*).

| **Species** | **Parameter covariate** | **Occupancy (Ψ)** | **Detection (*p*)** |
| --- | --- | --- | --- |
| **Leopard** | carnivore |  | **-0.56 (-0.83, -0.52)** |
|  | altitude |  | **-0.22 (-0.39, -0.16)** |
|  | water |  | **0.22 (0.04, 0.41)** |
|  | prey | -0.83 (-1.82, 0.16) | 0.17 (-0.03, 0.37) |
|  | habitation | **3.58 (1.22, 6.03)** |  |
|  | water source (seasonal) | **-3.88 (-7.07, -0.68)** |  |
|  |  |  |  |
| **Duiker** | leopard |  | 1.67 (-0.32, 3.66) |
|  | altitude | 3.83 (-0.77, 8.43) | **-12.58 (-18.56, -6.60)** |
|  | water |  | **2.68 (0.90, 4.45)** |
|  | vegetation age |  | **-9.66 (-15.04, 4.27)** |
|  | vegetation (Karoo) |  | -22.78 (-100.16, 54.60) |
|  | habitation |  | **-2.94 (-4.58, -1.29)** |
|  | road | -7.81 (-18.37, 2.76) | -5.49 (-11.13, 0.15) |
|  |  |  |  |
| **Grysbok** | vegetation (Karoo) |  | **-4.49 (-6.56, -2.43)** |
|  | road | **1.13 (0.10, 2.16)** | **-0.41 (-0.62, -0.19)** |
|  | disturbance (yes) |  | **1.49 (0.81, 2.16)** |
|  | leopard | 0.69 (-0.09, 1.47) |  |
|  | water source (seasonal) | -1.75 (-3.64, 0.13) |  |
|  |  |  |  |
| **Hyrax** | vegetation (Karoo) | **2.03 (0.45, 3.61)** | **0.88 (0.47, 1.29)** |
|  | road |  | **0.48 (0.23, 0.72)** |
|  | altitude |  | **-0.29 (-0.53, -0.05)** |
|  | carnivore | **-3.38 (-5.66, -1.10)** | -0.52 (-1.23, 0.19) |
|  |  |  |  |
| **Klipspringer** | vegetation (Karoo) |  | **0.72 (0.39, 1.04)** |
|  | disturbance (yes) |  | **-0.75 (-1.17, -0.33)** |
|  | carnivore |  | **-1.26 (-1.74, -0.77)** |
|  | water source (seasonal) | 1.31 (-0.09, 2.71) |  |
|  | altitude | **0.90 (0.05, 1.76)** |  |
|  |  |  |  |
| **Porcupine** | vegetation (Karoo) |  | **-2.00 (-2.78, -1.23)** |
|  | vegetation age | **0.68 (0.12, 1.24)** | **-0.51 (-0.73, -0.30)** |
|  | road |  | **-0.40 (-0.69, -0.11)** |
|  | carnivore |  | 0.18 (-0.01, 0.37) |
|  | water | 0.58 (-0.02, 1.18) |  |
|  |  |  |  |

Table S6: Top ranked models (*w* > 0.10 and QAICc < 2) for each species for Piketberg, using quasi-likelihood information criterion for small sample size (QAICc), delta QAICc (△QAICc), QAICc weight (QAICcwt) and the number of parameters (K).

| **Species** | **Model** | **K** |  | **QAICc** | **△QAICc** | **QAICcwt** |
| --- | --- | --- | --- | --- | --- | --- |
| **Leopard** | Ψ(null) *p*(vegetation + livestock) | 7 |  | 591.02 | 0.00 | 0.33 |
|  | Ψ(altitude) *p*(vegetation + livestock) | 8 |  | 591.48 | 0.46 | 0.26 |
|  | Ψ(water source) *p*(vegetation + livestock) | 8 |  | 592.67 | 1.65 | 0.14 |
|  | Ψ(livestock) *p*(vegetation + livestock) | 8 |  | 592.78 | 1.76 | 0.14 |
|  | Ψ(habitation) *p*(vegetation + livestock) | 8 |  | 592.97 | 1.95 | 0.12 |
|  |  |  |  |  |  |  |
| **Duiker** | Ψ(null) *p*(road + leopard + altitude + habitation) | 7 |  | 477.82 | 0.00 | 0.22 |
|  | Ψ(caracal) *p*(road + leopard + altitude + habitation) | 8 |  | 478.31 | 0.50 | 0.17 |
|  | Ψ(water) *p*(road + leopard + altitude + habitation) | 8 |  | 478.85 | 1.03 | 0.13 |
|  | Ψ(disturbance) *p*(road + leopard + altitude + habitation) | 8 |  | 479.30 | 1.48 | 0.10 |
|  | Ψ(vegetation) *p*(road + leopard + altitude + habitation) | 10 |  | 479.33 | 1.52 | 0.10 |
|  |  |  |  |  |  |  |
| **Grysbok** | Ψ(altitude) *p*(road + leopard + altitude + habitation) | 8 |  | 621.93 | 0.00 | 0.37 |
|  | Ψ(null) *p*(road + leopard + altitude + habitation) | 7 |  | 622.36 | 0.43 | 0.30 |
|  | Ψ(leopard) *p*(road + leopard + altitude + habitation) | 8 |  | 623.31 | 1.38 | 0.18 |
|  | Ψ(disturbance) *p*(road + leopard + altitude + habitation) | 8 |  | 623.71 | 1.78 | 0.15 |
|  |  |  |  |  |  |  |
| **Hyrax** | Ψ(caracal) *p*(leopard + caracal) | 6 |  | 398.75 | 0.00 | 0.37 |
|  | Ψ(caracal + leopard) *p*(leopard + caracal) | 7 |  | 399.55 | 0.83 | 0.25 |
|  | Ψ(caracal + habitation) *p*(leopard + caracal) | 7 |  | 399.60 | 0.88 | 0.24 |
|  | Ψ(caracal + leopard + habitation) *p*(leopard + caracal) | 8 |  | 400.50 | 1.78 | 0.15 |
|  |  |  |  |  |  |  |
| **Klipspringer** | Ψ(caracal + leopard) *p*(water + water source + road + caracal) | 9 |  | 381.72 | 0.00 | 0.17 |
|  | Ψ(caracal) *p*(water + water source + road + caracal) | 8 |  | 382.30 | 0.58 | 0.13 |
|  | Ψ( caracal + leopard) *p*(water + water source + road + caracal) | 10 |  | 382.47 | 0.75 | 0.12 |
|  |  |  |  |  |  |  |
| **Porcupine** | Ψ(caracal) *p*(habitation + disturbance + altitude) | 7 |  | 592.53 | 0.00 | 0.38 |
|  | Ψ(caracal + road) *p*(habitation + disturbance + altitude) | 8 |  | 592.93 | 0.40 | 0.31 |
|  | Ψ(caracal + disturbance) *p*(habitation + disturbance + altitude) | 8 |  | 592.99 | 0.45 | 0.30 |
|  |  |  |  |  |  |  |

Table S7: Model averaged covariate coefficient estimates (and 95% confidence intervals (CIs)) from the best approximating model from the analyses examining factors related to the space-use of species in Piketberg. Bold estimates indicate that there was a strong association (CIs do not overlap zero) between the covariate and species occupancy/space-use (Ψ) or detection (*p*).

| **Species** | **Parameter covariate** | **Occupancy (Ψ)** | **Detection (*p*)** |
| --- | --- | --- | --- |
| **Leopard** | vegetation (Renoster) |  | -0.11 (-0.82, 0.61) |
|  | vegetation (Riverine thicket) |  | 0.02 (-0.67, 0.72) |
|  | vegetation (Sandveld) |  | **2.10 (-3.59, -0.60)** |
|  | livestock (yes) |  | **0.62 (-1.14, -0.11)** |
|  | null | **2.46 (1.32, 3.59)** |  |
|  |  |  |  |
|  |  |  |  |
| **Duiker** | leopard |  | **-1.07 (-1.49, -0.66)** |
|  | altitude |  | -0.37 (-0.62, 0.11) |
|  | habitation |  | **-0.31 (-0.52, -0.09)** |
|  | road |  | **-4.18 (-5.82, -2.54)** |
|  | null | 0.20 (-0.44, 0.85) |  |
|  |  |  |  |
| **Grysbok** | road |  | **-0.28 (-0.55, -0.02)** |
|  | leopard |  | **0.26 (0.07, 0.45)** |
|  | altitude | 0.71 (-0.15, 1.57) | **0.42 (0.21, 0.63)** |
|  | habitation |  | **-0.24 (-0.47, -0.00)** |
|  |  |  |  |
| **Hyrax** | leopard |  | **-0.51 (-0.85, -0.16)** |
|  | caracal | **-1.72 (-3.05, -0.39)** | **1.18 (0.25, 2.10)** |
|  |  |  |  |
| **Klipspringer** | water source (seasonal) |  | **-1.18 (-1.81, -0.55)** |
|  | water |  | **-0.82 (-1.15, -0.49)** |
|  | road |  | **-0.37 (-0.66, -0.07)** |
|  | caracal | **-1.51 (-2.79, -0.23)** | **-0.96 (-1.67, -0.27)** |
|  | leopard | -0.62 (-1.34, 0.11) |  |
|  |  |  |  |
| **Porcupine** | habitation |  | **0.25 (0.06, 0.44)** |
|  | disturbance (yes) |  | **0.90 (0.17, 1.64)** |
|  | altitude |  | 0.18 (-0.01, 0.37) |
|  | caracal | **2.06 (0.01, 4.11)** |  |
|  |  |  |  |
